# Content-specific vulnerability of recent episodic memories in Alzheimer's disease

**DOI:** 10.1016/j.neuropsychologia.2021.107976

**Published:** 2021-09-17

**Authors:** Xenia Grande, David Berron, Anne Maass, Wilma A. Bainbridge, Emrah Düzel

**Affiliations:** a German Center for Neurodegenerative Diseases, Magdeburg, Germany; b Institute of Cognitive Neurology and Dementia Research, Otto von Guericke University Magdeburg, Germany; c Clinical Memory Research Unit, Department of Clinical Sciences Malmö, Lund University, Lund, Sweden; d Institute of Cognitive Neuroscience, University College London, United Kingdom; e Department of Psychology, University of Chicago, Chicago, IL, USA

**Keywords:** Episodic memory, Memorability, Memory representations, Medial temporal lobe, Alzheimer's disease

## Abstract

Endel Tulving's episodic memory framework emphasizes the multifaceted re-experiencing of personal events. Indeed, decades of research focused on the experiential nature of episodic memories, usually treating recent episodic memory as a coherent experiential quality. However, recent insights into the functional architecture of the medial temporal lobe show that different types of mnemonic information are segregated into distinct neural pathways in brain circuits empirically associated with episodic memory. Moreover, recent memories do not fade as a whole under conditions of progressive neurodegeneration in these brain circuits, notably in Alzheimer's disease. Instead, certain memory content seem particularly vulnerable from the moment of their encoding while other content can remain memorable consistently across individuals and contexts. We propose that these observations are related to the content-specific functional architecture of the medial temporal lobe and consequently to a content-specific impairment of memory at different stages of the neurodegeneration. To develop Endel Tulving's inspirational legacy further and to advance our understanding of how memory function is affected by neurodegenerative conditions such as Alzheimer's disease, we postulate that it is compelling to focus on the representational content of recent episodic memories.

## Highlights

• The functional anatomy of episodic memory segregates different memory content.
• Alzheimer's disease may cause content-specific loss of recent memories
• Content-specific memorability across individuals changes with Alzheimer's disease.
• Content-specific assessment could provide new insights into episodic memory in health and disease

“A hundred years ago, memory was a simple and well-understood faculty of the brain/mind, and it was easy to talk and write about it and its pathology with authority. Thanks to all the research that has been done since that time, memory today is no more simple nor is it well understood.”

(Tulving, 1997)

Endel Tulving's conceptual distinction of episodic and semantic memory marks a major advance in memory research. It has remained hugely influential in basic and clinical research aimed to unravel the neural processes that allow experiences to be remembered. In his insightful and still topical definition, Tulving stated that, “Episodic memory receives and stores information about temporally dated episodes or events, and temporal-spatial relations among these events.” (Tulving, 1972). Thereby episodic memory is distinct from semantic memory, which he described as “a mental thesaurus [that] organize[s] knowledge a person possess[es]” (Tulving, 1972). This definition of episodic memory as a faculty that captures rich, multi-modal personal events in coherent recollective experiences has stood the test of time. Throughout this paper we will show how Tulving's episodic-semantic framework has guided the evaluation of recent memories in research and clinical settings. We argue that it is timely to develop the assessment of memories based on their experiential nature further towards a focus on the explicit content that a memory represents in order to understand how memory function is affected by disease.

Indeed, the coherent experiential nature of episodic memory is an important component of Tulving's theory which he developed further in the 1980's and 1990's (Düzel et al., 1997; Tulving, 1985; Schacter and Tulving, 1994). He posited that episodic memory is governed by a particular type of conscious awareness of information about previously experienced events: autonoetic awareness (Tulving, 1985; Wheeler et al., 1997). “It is the kind of awareness that characterizes mental ‘re-living’ of happenings from one's personal past. It is phenomenologically known to all healthy people who can ‘travel back in time’ in their own minds.” (Düzel et al., 1997). In contrast, noetic awareness (knowing) accompanies an individual's interaction with its environment in the present.

This concept led to a particular form of memory assessment in research and clinical diagnostics, namely the Remember/Know paradigm (Gardiner, 1988; Tulving, 1985). A major support for Tulving's concept came from clinical observations of impaired memory for personal experiences but preserved memory for learned facts. Notably, patients with developmental amnesia showed a striking impairment of episodic memory while semantic memory appeared to be intact (Gardiner et al., 2008; Vargha-Khadem et al, 1997, 2001). Impaired autonoetic awareness of events was reflected in the inability to remember, that is mentally ‘re-live’ information, and the rather selective impairment of neural signatures reflecting remembering (Düzel et al., 2001).

The proposition of a coherent experiential nature underpinning episodic memory permeates how scientists and clinicians evaluate its impairment. This is illustrated in the fact that the diagnostic assessment of episodic memory is performed largely independent of the representational content of memoranda (Costa et al., 2017). Tests for memory function entail a large variety of different types of stimuli, administered in many different tasks requiring some form of recollection or recall. Faces, words and images, visual and auditory as well as story content can be found as memoranda in different standard tests, as for example the “Doors and People Test” (Baddeley et al., 1994), the “Verbal Learning and Memory Test” (Helmstaedter and Durwen, 1990), the Wechsler Memory Scale (Wechsler, 1987), the “Face – Name Associative Memory Exam” (Rentz et al., 2011) or the “Free and Cued Selective Reminding Test” (Buschke, 1984). In practice, any of these tests are employed to evaluate memory function, with the tests being agnostic for the assessed content (Costa et al., 2017). In our view, this reflects an overarching conception in neuropsychological assessment that when episodic memory is impaired, the ability to remember recent events equally fades for all types of information due to their convergence in a multimodal processing hierarchy (Costa et al., 2017; Mishkin et al., 1998). Memory function is thus evaluated so far without careful consideration of the to-be-remembered material. Meanwhile, however, another possibility is emerging, namely that impaired episodic memory can be associated with a discrete loss of specific representations.

According to this idea, in a progressive neurodegenerative condition such as Alzheimer's disease, the ability to remember certain event content could fade before other content is affected. Especially in early stages of the disease when the impairment is not yet complete, an accumulating body of research suggests that individuals may have preserved memory for certain representations. The intriguing possibility is that the content type of this selective impairment may be hard-wired into the anatomy of episodic memory and therefore constant across individuals and situations. This alternative conception of episodic memory impairment has been barely considered so far.

Before we develop our view, we want to explicitly point out that our proposal refers to recent episodic memories, thus memories before systems consolidation takes place. We focus on a phenomenon that takes place during encoding and presumably early molecular synaptic consolidation (Lisman et al., 2011), thus in the early hours of a memory trace. As such, our current opinion paper does not address autobiographical memories that define a person's biography and the memory profile a person with Alzheimer's dementia still experiences about the personal past. These autobiographical memories are amalgamates of episodic, semantic and personal semantic information that have been acquired in the past (Kopelman et al., 1989). Research on autobiographical memories has had a long-standing focus on content-specific aspects of memory (cf. Kopelman et al., 1989; Levine et al., 2002). The contents of autobiographical memory traces are shaped by hippocampal-neocortical or neocortical-neocortical interactions and re-consolidation, ensuing content-specific vulnerability and stability (Moscovitch et al., 2005; Nadel et al., 2000; Nadel and Moscovitch, 1997; Winocur and Moscovitch, 2011). While we acknowledge that these systems consolidation processes may well be influenced by the initial shape of a memory trace, they are not the focus of our current proposal. Here, we aim to increase awareness for the phenomenon of content-specific vulnerability of episodic memories apparent shortly after encoding and prior to systems-level consolidation processes.

In that light, we discuss the possibility that fading memories may affect certain representations more strongly than others, creating “islands of relatively intact recollection” (note, this term has been used in relation to remote memory impairment in transient epileptic amnesia e.g. by Butler and Zeman, 2008) whose representational building bricks are consistently reproducible across individuals. In the following, we first highlight aspects of the functional architecture of episodic memory that show how specific memory content is processed in the brain. We will illustrate the vulnerability of episodic memories as the leading symptom in acute hippocampal injuries causing amnesia and progressive conditions such as Alzheimer's disease. We continue to review the recent attempts in advancing the classical investigation and description of episodic memory in terms of experiential nature and processes focusing on the content of episodic memories. We present recent insights into a high level of consistency across episodic memories in their likelihood to be remembered—regardless of the observer and the situation, certain memories are intrinsically more *memorable* than others. We will show how the functional architecture of episodic memory and memorability may relate to each other and conclude by discussing the implications for future research and our understanding of impaired memory.

## 1 Recent insights into the functional architecture of episodic memory

For decades, researchers aimed to identify the processes that underlie the formation and experiential nature of episodic memory and unravel which brain structures give rise to our awareness of past experiences. In the hippocampus, multiple information processing streams converge, rendering it essential to create and relive a coherent memory of rich multimodal events (Mishkin et al., 1998). The holistic experience of episodic memory is however not only accomplished by medial temporal lobe structures but by a widespread network of interacting brain regions that also spans frontal and parietal cortices (Cabeza et al., 1997; Nyberg et al., 1996a,b; Nyberg et al., 1996a,b; Nyberg et al., 2001, 2000; Simons and Spiers, 2003; Wagner et al., 2005).

One major recent achievement in understanding the functional architecture of episodic memory is the refinement of the structure-function mapping within the hippocampus. The hypothesis that anatomical features of subfields within the hippocampal circuitry map onto different memory processes (Marr, 1971) had already been formulated at around the same time that Tulving introduced the episodic memory concept. However, only recently have advances in high resolution neuroimaging allowed the field to study hippocampal subregions functionally in humans.

When a new episodic memory is to be formed, information that belongs to the current event needs to be associated and integrated into a coherent memory representation while at the same time being kept separate from representations of other past experiences. Access to these multi-element memories must be triggerable by cues that represent only a fraction of the original event. To assure both the separation and the integration of mnemonic information, a recurrent information flow between hippocampus and cortex has been proposed (Koster et al., 2018; Kumaran and McClelland, 2012). Within the hippocampus, the subregions dentate gyrus (DG), CA3 and CA1 act via distinct mechanisms on incoming information. In DG, a pattern separation mechanism distinguishes similar inputs into distinct representations (Berron et al., 2016; Leutgeb et al., 2007; Neunuebel and Knierim, 2014). In CA3, however, a pattern completion mechanism completes a partial memory cue to previously stored full representations (Grande et al., 2019; Nakazawa et al., 2002; Neunuebel and Knierim, 2014). The completed representation is then transferred to CA1 where it can interact with incoming information. This interaction may be akin to a comparator (e.g. Hasselmo et al., 1996; Lisman et al., 2011) determining whether the incoming information is novel or old and thus gets stored in a new, distinct representation, or gets reinstated in cortical areas for a full recollective experience (Bartsch et al., 2011; Chen et al., 2011; Dimsdale-Zucker et al., 2018; Duncan et al., 2012; Maass et al., 2014; Schlichting et al., 2014). The anatomical organization of cortical reinstatement, in turn, seems to depend on the sensory domain or content of information (Cabeza et al., 1997; Horner et al., 2015; Nyberg et al., 1996a,b; Nyberg et al., 2000). The perspective of structure-process mapping thus revealed how multifaceted memory representations become differentiated from one another and how the experiential nature of episodic memory may relate to cortical reinstatement.

However, besides the content-specificity in cortical reinstatement, the process-oriented perspective on memory makes no structural distinction for various types of information. This view is challenged by representation-based models, in which mnemonic content co-determines the anatomical anchoring in the brain. Thus, one recent achievement in understanding the functional architecture of episodic memory is the consideration of a structure-*content* mapping. It originates in the debate about mapping mnemonic experiences, i.e. familiarity versus recollection (related to Remember/Know but also see Gardiner, 2001 for a more differentiated view) to structures in the medial temporal lobe. Initially, dual-process accounts interpreted reports about patients with hippocampal lesions but preserved recognition ability as evidence for a functional dissociation between the perirhinal cortex and hippocampus in underpinning familiarity versus recollection experiences (Yonelinas et al., 2005). Familiarity, in this context, described retrieval based on a general sense of knowing whereas retrieval via recollection entailed remembrance of the context in which the memory was acquired. A fundamentally different perspective on functional dissociations within the medial temporal lobe was however taken by Eichenbaum and later by Graham, Ranganath and colleagues (Eichenbaum, 2000; Graham et al., 2010; Ranganath and Ritchey, 2012). Their interpretation of clinical data considered the represented informational content, where the perirhinal and parahippocampal cortices are associated with item versus contextual content while the hippocampus is thought to bring both streams together. The posterior medial-anterior temporal framework (Ranganath and Ritchey, 2012; Ritchey et al., 2015) expanded the initial idea to a larger network perspective that segregates the whole brain into two different information processing pathways. Context information is processed via the posterior medial system (connecting the retrosplenial cortex, the angular gyrus, precuneus, posterior cingulate and the parahippocampal cortex) while item information is processed mainly via the anterior temporal system (connecting the perirhinal cortex and the amygdala, the anterior ventral temporal cortex and lateral orbitofrontal cortex; for evidence see for example Reagh and Yassa, 2014; Berron et al., 2018). Irrespective of the task at hand being rather perceptual or mnemonic, item stimuli like objects and faces were thus considered to be preferentially processed in the perirhinal cortex (and connected structures of the anterior temporal system) whereas context stimuli like scenes were considered to be preferentially processed in the parahippocampal cortex (Lee et al., 2005; Liang et al., 2013; Litman et al., 2009; Ross et al., 2016; Staresina et al., 2011). This representational segregation is indicated also within the entorhinal cortex and the hippocampal transversal and longitudinal axis with the anterior-lateral and posterior-medial entorhinal cortex continuing the anterior temporal system and posterior medial system, respectively (Knierim et al., 2014; Maass et al., 2015; Navarro Schröder et al., 2015) and in turn building representational streams with the proximal and distal subiculum, distal and proximal CA1 and presumably transversal sections of CA3 (Beer et al., 2018; Flasbeck et al., 2018; Henriksen et al., 2010; Nakamura et al., 2013; Nakazawa et al., 2016; Ng et al., 2018; Sun et al., 2017, 2018). Along the longitudinal axis of the hippocampus, a gradient from coarser anterior representations towards finer posterior representations has been reported (Small, 2002; Poppenk et al., 2013; Strange et al., 2014; Brunec et al., 2018). Note, however, that representational content is not completely dissociable into two different types (i.e. context versus item, global versus local or spatial versus non-spatial) between the anterior temporal and the posterior medial system. Ample projections between subregions exist and only recently it has been shown that the parahippocampal cortex projects to both subregions of the rodent entorhinal cortex and not as initially thought, to the medial entorhinal cortex exclusively (Doan et al., 2019; Nilssen et al., 2019). Nevertheless, a bias to process certain types of information within specific structures is evident across species, even if the informational content may merge at various locations throughout the processing hierarchy in the medial temporal lobe

To briefly summarize, the understanding of how memories emerge in the brain is currently advancing by a more fine-grained structure-process mapping in the medial temporal lobe and a focus on the interplay of functionally heterogeneous subregions. In addition, recent investigations acknowledge that content may be inherent to the specific functional architecture that gives rise to memory. New accounts consider interactions between these process- and content-oriented approaches to understand episodic memory function and acknowledge that systems storing specific types of representations may be shaped by computational operations that are executed in certain subregions (e.g. Bastin et al., 2019; Cowell et al., 2019; see also Ekstrom and Yonelinas, 2020). Future research may show how certain computations require and shape specific representations (e.g. pattern separation operations on conjunctive representations) and thereby reveal whether and how process- and content-oriented accounts are intrinsically related.

The new insights into the functional architecture from which episodic memory emerges are particularly exciting when we aim to understand impairment of episodic memory in disease conditions. Notably, it changes the traditional way of assessing the vulnerability of recent episodic memories as we illustrate in the following paragraphs.

## 2 Episodic memory impairment after acute brain injury

Clinical research into the nature of impaired episodic memory after acute brain injury has focused on the question of whether its impairment can be selective and dissociated from relatively intact semantic memory. This research utilized direct assessments of semantic and episodic details in memory functions and also indirect approaches using autonoetic and noetic awareness as proxies for episodic and semantic memory. We will briefly refer to this literature before moving on to impaired episodic memory in progressive neurodegenerative conditions, such as Alzheimer's disease and then to memorability and content-specific impairment patterns within episodic memory.

While patient H.M. provided the first prominent evidence for the role of the medial temporal lobe and hippocampus in explicit memory, K.C. provided evidence for the semantic-episodic memory distinction. The profile of memory impairment in patient K.C. was striking. His episodic memory was severely impaired while as in H.M., his general intellectual capacity was normal and he was unimpaired in tasks that required a mere knowledge-based usage of memories (Milner et al., 1968; Rosenbaum et al, 2005, 2012). In K.C., new learning of semantic information was explicitly tested and while it was slow, it was found to be possible (Rosenbaum et al., 2005). Both, K.C. and H.M., developed medial temporal lobe lesions later in life caused by traumatic brain injury and surgical brain damage, respectively. A groundbreaking study with three cases of developmental amnesia, however, confirmed that episodic memory can be profoundly impaired while semantic memories can be acquired, even giving amnesic individuals the possibility to attend school with an average range of success (Vargha-Khadem et al., 1997; but see Squire and Zola, 1998 for another interpretation). A main focus of investigations thus became the experiential nature of memories in these and other amnesic patients. Clear evidence for a specific impairment in autonoetic consciousness upon retrieval was provided with the developmental amnesia patient Jon, who showed electrophysiological and behavioral responses compatible with a sense of knowing despite a lack in the experience of recollection and associated electrophysiological signatures (Düzel et al, 1997, 2001). Also more recently, a lack of autonoetic consciousness was shown in a study with 16 patients that suffered from lesions in hippocampal subregion CA1 and transient global amnesia (Bartsch et al., 2011).

These amnesia cases show that a decline in awareness may be present but other aspects of a memory may still be preserved (see also Düzel et al., 2001). It is essential to note, however, that the location of lesions is very heterogeneous from patient to patient and so is the profile of the memory impairment. When the hippocampus is spared, for example, recollection seems to be intact while semantic memories are affected (e.g. Bowles et al., 2007).

## 3 Episodic memory impairment in progressive neurodegeneration: Alzheimer's disease

Unlike acute brain injury, Alzheimer's disease is associated with a progressive and relatively stereotypic memory decline across individuals. The most prominent risk factor for Alzheimer's disease is old age and, indeed, the aging brain is already subject to widespread neural changes (Buckner, 2004) including frontal (Andrews-Hanna et al., 2007; Daselaar and Cabeza, 2008; Davis et al., 2008) and medial temporal lobe (Leal and Yassa, 2015; Raz et al., 2005) regions. Countless studies have investigated which aspects of memory change with age and which specific processes deteriorate. Besides a prominent impairment in executive components of memory (Shing et al., 2008; Daselaar and Cabeza, 2008), aging generally affects the richness of remembered information and also the ability to bind multiple elements in memory (Levine et al., 2009; Old and Naveh-Benjamin, 2008; St. Jacques et al., 2012; Yonelinas et al., 2007; Piolino et al., 2006; St. Jacques and Levine, 2007). A difficulty to separate memory representations from each other (Reagh et al, 2015, 2018; Yassa et al, 2011a, 2011b) may go hand in hand with a bias to pattern complete memory cues (Vieweg et al., 2015), resulting in false “memories” (Devitt and Schacter, 2016; Fandakova et al., 2015). While episodic memory is strongly susceptible to decline with age, semantic memory is less affected (Zacks et al., 2000). This leads to a profile of fragmented autobiographical memories, still preserving semantic details and personal semantics while lacking episodic content like personal thoughts (e.g. Levine et al., 2002; Piolino et al., 2002).

The two hallmark pathologies underlying Alzheimer's disease are neurofibrillary tangles and beta-amyloid plaques (Braak and Braak, 1991, 1995, 1991; Braak and Del Trecidi, 2015; Hyman et al., 1989; McKhann et al., 2011). Both are anatomically progressive pathologies with stereotypic spreading patterns in the brain. In human imaging studies, amyloid pathology frequently begins in medial parietal structures, including retrosplenial cortex, posterior cingulate and precuneus as well as medial frontal areas (Grothe et al., 2017; Mattsson et al., 2019; Palmqvist et al., 2017; Villeneuve et al., 2015). Cortical tau pathology, in contrast, frequently begins in the transentorhinal area before spreading to the entorhinal cortex, parts of the hippocampus, then the perirhinal cortex, the lateral temporal lobe and finally cortical frontal and parietal regions (Braak et al., 2006; Braak and Braak, 1991).

The pathology, ultimately concomitant of cell loss in the respective brain regions, affects brain regions that are crucial for successful episodic memory (Jagust, 2018; Jagust et al., 2006). Accumulating evidence shows that tau pathology in the medial temporal lobe best predicts episodic memory decline (e.g. Maass et al., 2017; Lowe et al., 2018; Sperling et al., 2019; Hanseeuw et al., 2019) while amyloid-burden alone shows only weak associations to episodic memory performance. However, more rapidly progressive memory decline is most likely when both types of pathology converge (Betthauser et al., 2019). Whether indeed only the synergistic effect of both pathologies leads to progressive memory decline is still under investigation (Jessen et al., 2014; Lowe et al., 2018; Maass et al., 2017; Schöll and Maass, 2020; Sperling et al., 2018).

The early stages of Alzheimer's disease are associated with episodic memory impairments while semantic processing has been found intact (Morris and Kopelman, 1986). Research points towards increasing reliance on semantic details and gist memory in these stages (El Haj et al., 2017 for an overview). A temporal gradient has been frequently reported with preserved remote memories but impaired formation and retrieval of new memories (Irish et al., 2011a,b; Irish et al., 2011a,b; Kopelman et al., 1989; McKhann et al., 2011; Addis and Tippett, 2004). Content-specific assessment of autobiographical memories however indicates that the temporal gradient could be more pronounced for (personal) semantics while episodic components are impaired throughout (Irish et al., 2011a,b; Piolino et al., 2003), a finding that may depend on the specifics of the assessment method (Barnabe et al., 2012). Moreover, recent preliminary findings show particular impairment on everyday memory under conditions of delayed recall and for associative memories in mild cognitive impairment (MCI, Irish et al., 2011a,b). Interestingly, however, certain rich cues, for instance music, odors or pictures (e.g. El Haj et al., 2020, 2012; El Haj et al., 2018) may still evoke fragmented autobiographical memories and memories may be enhanced by a focus on self-referential aspects (e.g. Carson et al., 2019; El Haj and Antoine, 2017a; Kalenzaga et al., 2013) as well as with emotional cues in early disease stages (e.g. Hamann et al., 2000; Kensinger et al., 2004; Kumfor et al., 2013; Sava et al., 2015). Overall, emotional components of a memory seem preserved despite a general diminished sense of reliving and visual imagery in Alzheimer's dementia (El Haj et al., 2016; El Haj and Antoine, 2017a; Rauchs et al., 2007). Indeed, extensive investigations of autobiographical memories (Levine et al., 2002) carved out a specific profile and revealed that within a single remembered autobiographical episode, Alzheimer's dementia is related to specific impairment of the event-related and personal thought related details, and a bias to report more semantic details (Barnabe et al., 2012; Irish et al., 2011a,b; Murphy et al., 2008).

Regarding recent episodic memory, some studies show particularly impacted free and delayed recall (e.g. Bäckman et al., 2005), while a meta-analysis finds recognition to be only preserved in preclinical Alzheimer's dementia (MCI) but not with progressed states of the disease (Koen and Yonelinas, 2014). Comparable to autobiographical memories, it additionally became evident recently that among recent episodic memories, certain material shows more vulnerability for memory impairment. Hence mnemonic discrimination for item information is more impaired than scene information with beginning tau pathology (Berron et al., 2019; Maass et al., 2019). This observation sets the stage for investigations on the diagnostic value and specific memorability of certain content within episodic memory with various levels of pathology in the early stages of Alzheimer's disease (Bainbridge et al., 2019a).

Note that the pathology is evident more than a decade before the first clinical symptoms develop (Braak and Braak, 1991; Ossenkoppele et al., 2019). About 30 % of seemingly healthy individuals over 65 years of age bear “hidden” amyloid pathology, whereas more than 60 % of elderly people show tau pathology in the medial temporal lobe (Braak and Braak, 1997) and cognitive alterations are not necessarily detectable. At which point age-related and pathological Alzheimer's processes lead to differential profiles in episodic memory decline has yet to be determined (Jack et al., 2010).

Irrespective of the debate about the neuropathological distinction between normal aging and Alzheimer's, diagnostic assessments of memory function in old age and Alzheimer's disease consider episodic memory as a content-independent clinical symptom. Thus, similarly to research on amnesia, the main focus is on the experiential nature of fading memories and the processes that are affected. The recent insights into the functional architecture of episodic memory, however, also highlight memory content as an important variable in the evaluation of episodic memory decline.

## 4 The memorability of episodic memories

Memorability refers to the observation that irrespective of the testing situation and consistently across individuals, some stimuli are more likely to be remembered than others (Bainbridge et al., 2013; Isola et al., 2011). This memorability of a stimulus has been shown to account for as much as 50 % of the variance in memory performance (Bainbridge et al., 2013), and is consistent across different tasks, image contexts, presentation and retention times (Broers et al., 2018; Bylinskii et al., 2015; Goetschalckx et al., 2018). Independent of attention, priming effects or top-down influences, the phenomenon is considered to be “automatic” (Bainbridge, 2020), determined already 160 ms after stimulus onset (Mohsenzadeh et al., 2019) and related to functional activity in late visual regions (inferotemporal cortex), the medial temporal lobe and the anterior hippocampus (Bainbridge et al., 2017; Bainbridge and Rissman, 2018; Jaegle et al., 2019).

Memorability thus appears to be an inherent feature of episodic memory. Current research seeks to identify the qualities and the specific content that determines how memorable an episode is likely to be. While several attributes for an image have shown correlations with memorability, no singular attribute has been found that can act as a proxy for memorability. For example, manmade scenes containing many objects tend to be more memorable than outdoor natural scenes (Bainbridge et al., 2019a; Isola et al., 2014), however these attributes do not explain a high amount of variance in memorability. Low level qualities like color coding or brightness and also the eye fixation time during encoding seem not to be able to explain an image's memorability (Bainbridge et al., 2013; Bainbridge et al., 2019a; Isola et al., 2011). Other high-level qualities of an image such as its aesthetics, emotional content, and even observer's own ratings of how memorable an image appears do not show strong correlations with memorability (Bainbridge et al., 2013; Isola et al., 2014). Recent work utilizing computational models and neuroimaging techniques have suggested that above all, it may be the composition of the elements an episode consists of, in particular an item's relationship to other items in the representational space of a memory that influences an episode's memorability. For example, research using deep learning methods has found that more sparsely distributed items are more memorable (Lukavský and Děchtěrenko, 2017), and that dissimilarity in low-level visual information may map onto memorability (Koch et al., 2020). At the same time, similarity at the level of conceptual information may relate to memorability (Koch et al., 2020). For instance, highly semantically connected words are more memorable and are reinstated earlier in the anterior temporal lobe (Xie et al., 2020) and memorable images show more similar representational patterns in the brain than forgettable images (Bainbridge et al., 2017; Bainbridge and Rissman, 2018). An understanding of the principles that govern the memorability of an episode could reveal the computations performed after perceiving the episode that lead to successful memory encoding.

The memorability feature of episodic memories is especially compelling when it comes to the evaluation of memory decline. A recent behavioral study investigated memorability of photographic images in older adults that were either cognitively normal without memory complaints, cognitively normal but with a subjective memory decline severe enough to seek medical advice (subjective cognitive decline) or with significant (1.5 SDs) memory decline relative to the expected performance in old age (MCI) and showing a profile typical of prodromal Alzheimer's disease (Bainbridge et al., 2019a). If episodic memory decline from cognitively normal older adults to those with MCI would affect episodic memory irrespective of the representational content of photographic images, the outcome of this study would have been a reduced memory performance proportionally across all images. However, this study observed an asymmetry across images as related to memorability—a specific set of images remained highly memorable to cognitively normal adults but became forgettable to those with MCI. Looking at memory performance for these images specifically, we could significantly predict whether an individual suffers from MCI, better than any other set of images. Equally intriguingly, some stimuli remained consistently and highly memorable across healthy controls and MCI patients, and performance for those images could be predicted by deep learning models. Thus, while some stimuli seemed to be memorable across everyone (no matter the pathological condition), other stimuli seemed to be of diagnostic value as they were highly forgettable by individuals facing conditions of preclinical dementia but not by healthy controls (Bainbridge et al., 2019a). These results indicate that certain neural pathways essential for memory processes or for representing mnemonic information may be affected earlier in the course of decline than others, resulting in a specific pattern of episodic forgetting and potential islands of recollection. As we define them, these islands refer to certain mnemonic content that remains accessible to episodic memory when other types of information cannot be remembered anymore. Importantly, deliberate selection of the content to be remembered can promise to unveil these differences across neural pathways, and across different stages of cognitive decline.

Indeed, as briefly stated above, functional imaging in older adults show that the anterior temporal-posterior medial system segregation is less clear than in young adults and they recruit the anterior temporal system less (Berron et al., 2018). Increased tau pathology is related to a specific decrease in memory performance for object content, while scene content is preserved (Berron et al., 2019). This content-driven difference is also reflected in the observation that manmade scenes with multiple objects are the first to show strong differences in memorability between healthy adults and those with MCI (Bainbridge et al., 2019a). In general, accumulating evidence shows that tau pathology affects anterior temporal regions and possibly isolates the hippocampus from the large-scale anterior temporal network while amyloid leads to a deficit in the posterior medial network function (Adams et al., 2019; Harrison et al., 2019). This may explain content-specific memory impairments in accordance with the preferentially processed information in the affected network (Maass et al., 2019; Berron et al., 2019; and see Berron et al., 2020 for effects on respective network connectivity). Thus far, while decodable patterns of memorability have been observed in parts of the anterior temporal network such as the perirhinal cortex and anterior temporal lobe, it is less clear whether posterior medial regions show information about the memorability of a stimulus (Bainbridge et al., 2017; Bainbridge and Rissman, 2018). However, other research has shown decodability of other memory content such as the identity or representational content of a memory from posterior medial regions like the retrosplenial and parahippocampal cortex (Bainbridge et al., 2021; Silson et al., 2019).

Initial findings thus point towards a potential relationship between the inherent feature of memorability based on memory content, an underlying functional architecture of biased information-processing in certain brain systems and content-specific memory decline in relation to pathology within these brain systems. An open question however will be how different types of memory content may allow researchers to pinpoint representational differences in the respective brain systems, and how performance on specific stimuli is associated to brain pathologies in certain functional networks.

## 5 Discussion and future perspectives

While we are still in the early days of understanding memorability, the phenomenon provides an intriguing new way on how we conceptualize episodic memory and interpret and investigate fading episodic memory. The observations that certain recent memories fade more easily than others across people, that the content and composition of episodic memories may drive their memorability, and that the content of episodic memories determines the specific underlying functional architecture, call for a change of perspective in how we investigate and evaluate episodic memory decline. We postulate that, to understand episodic memory function, we need to develop Tulving's legacy further and understand how content influences episodic memories and why this influence is hard-wired to the human brain so as to render it stable across individuals.

To illustrate these considerations, we refer to memories as “landscapes” which are affected by erosion. As much as a landscape is defined by the sum of its elements (i.e. mountains and forests), an episodic memory is defined by the sum of different types of information defining the event. Components of the landscape will have different vulnerabilities to erosion; trees and soil are likely to be affected much earlier than mountains composed of granite. Likewise various causes of erosion (e.g. continuous wind, rain, tornados or flooding) exert different forces on the components of a landscape. Similarly, healthy aging processes cause certain memory components to decline while pathological processes may excel these and even carve out a unique shape of the memory landscape. This analogy may help us to conceptualize how the landscape of memories may be affected in disease. The current insights into the functional architecture of the medial temporal lobe also suggest that different types of information are processed and represented in different neural pathways within the episodic memory network. Similarly, we think that islands of recollection in the memory landscape of episodic memory may prevail until later stages of neurodegeneration. Thus, rather than speaking of loss or impairment of episodic memory as a whole, it may be more appropriate to consider the possibility of impoverished episodic memory with selective loss of specific content.

The illustration of memory landscapes in episodic memory serves to highlight how our understanding of memory is shaped by how we test for episodic memory. If clinical research is guided by a model of episodic memory as a representationally-independent faculty of reliving past events, our discoveries and understanding will remain limited to what the model permits. If clinical research embraces the representational nature of episodic memory decline and assesses remembrance of recent episodic memories for different types of content, we may gain new insights into the episodic memory experience of patients with Alzheimer's disease.

The potential in investigating progressive impairment of recent episodic memories in neurodegenerative conditions is twofold. First, islands of recollection could provide a unique window into the organization of memory. Content-specific cognitive readouts could provide insights into which aspects of episodic memory are neuroanatomically distinct. In analogy to the early insightful observations on differences in systems consolidation for rather semantic versus rather episodic elements, also the observations on memorability obtained thus far are a first proof of concept that motivate further investigation. The second potential is of a clinical nature. Islands of recollection could be content-specific for certain stages of disease progression thereby enabling tailored tests for diagnostic staging. Moreover, specific strategies may allow us to harness preserved memory abilities to support activities of daily living.

What happens in the early hours of a memory trace that determines its memorability is entirely up to speculation for now. The novelty of memorability findings does not allow yet any firm hypotheses on mechanisms that drive variations in memorability within the population as well as between healthy older adults and older adults with Alzheimer's disease. We observe, however, that several aspects may play a role that are related to the way content-information is bound together, represented and sorted at encoding. One mechanism that determines memorability may be the level of integration within an item's representation. A word's memorability, for example is determined by its centrality in the semantic space (Xie et al., 2020). Highly memorable item representations may thus closely incorporate multiple features. Likewise, the inherent multimodality of certain content representations may drive memorability. For instance, in contrast to scenes, the representation of isolated objects is intrinsically multimodal, integrating olfactory, gustatory, auditory or tactile information. Note, that also among different objects, the level of multimodality changes (consider for instance a lamp versus a cup of coffee). Hence the involved functional architecture differs for multimodal objects and scene memories due to biased pathways of information processing (cf. Fiorilli et al., 2021; Lee et al., 2021; but note the described profound overlap as well). Under healthy conditions, multimodal representations may enhance memorability because a memory can be accessed via multiple ways (e.g. the cup of coffee via a scent or a taste). In Alzheimer's disease particularly multimodal representation areas like the perirhinal cortex (cf. Fiorilli et al., 2021; Bussey et al., 2005; Lee et al., 2021) are affected early on, presumably leading to an increased vulnerability for certain object memories. Another appealing mechanism that may determine differential memorability effects in Alzheimer's disease are attentional and perceptual mechanisms. When a stimulus consists of multiple objects, a condition may appear that potentially resembles simultanagnosia, that is the inability to perceive and bind multiple objects together while their single recognition is unaffected (Chechlacz et al., 2012; Coslett and Saffran, 1991). Whenever there is competition between multiple objects in complex scenes, attention-based deficits may be possible that hinder the binding of mnemonic elements, in particular when Alzheimer's pathology invades key object-processing structures along with the visuospatial attention system in posterior brain areas (Chechlacz et al., 2012) as one would typically expect in MCI. Consequently, memorability under Alzheimer's disease may be affected for isolated as well as multiple objects, but potentially being even more impaired for the processing of multiple objects in those with Alzheimer's disease in comparison to healthy individuals. Note that deliberately manipulating overall attention to a stimulus did not change memorability (Bainbridge, 2020), hence we are here referring to attentional dynamics driven by the stimulus itself. Overall, we think that memorability may reflect the order in which perceptual inputs are prioritized for memory encoding (cf. Xie et al., 2020), but future studies need to reveal whether this idea holds and unravel the mechanisms by which this prioritization takes place, potentially leading to different levels of integration within an item's representation.

Indeed, careful inspection of the memorability findings so far reveals that the above stated mechanisms may not be the full story and need further elaboration. First, our ideas may predict that in particular highly multimodal items like objects are memorable across people. However, single objects can be among forgettable items as well (Bainbridge et al., 2019a) and memorability effects have also been observed in abstract noise stimuli (Lin et al., 2021). Second, complex images containing multiple objects seem to be highly diagnostic, presumably driven by deficits in medial temporal lobe structures and attentional deficits (Bainbridge et al., 2019a). However, not all diagnostic images are cluttered and display a complex assembly of objects (Bainbridge et al., 2019a). Third, the finding that a lack of memorable qualities (esthetics, interest) leads to the forgetting of otherwise highly memorable objects under Alzheimer's dementia may follow from competition between items. However, studies on memorability that looked at many singular properties for predicting memorability (e.g. the number and size of objects, esthetics, interestingness etc., see e.g. Bainbridge et al., 2013; Bainbridge et al., 2019a; Isola et al., 2014) were not able to predict large variance in memorability (at least for faces as in Bainbridge et al., 2013). Presumably, our general representations about the larger visual statistical world (innate or learned) play an essential role. Thus, overall the intrinsic, task- and experience-independent nature of memorability is not yet fully explainable and still remains a secret of the brain's functional architecture.

Note that not only are many aspects of memorability still unresolved, but we also lack a full understanding of many aspects concerning the functional architecture of the medial temporal lobe and the specificities of Alzheimer's pathology progression. For instance, there have been general reports of left hemispheric lateralization in the processing of verbalizable, semantic material (Dalton et al., 2016; Golby et al., 2001; Kennepohl et al., 2007). In addition there are recent findings that imply more profound effects of Alzheimer's pathology in the left hemisphere, at least for some subtypes (Berron et al., 2020; Ossenkoppele et al., 2016; Vogel et al., 2021). How these dynamics interact during the formation of memories and how they are reflected in the memorability of items is open for further research.

Besides the multidimensionality of episodic memories, a core quality of episodic memory according to Tulving is the autonoetic nature. Some even consider autonoetic consciousness an essential prerequisite of memory (Klein, 2015; Klein and Markowitsch, 2015). A key question that needs to be addressed when considering memorability is to what extent preserved memorability is associated with autonoetic awareness. It may be possible that, similarly to some preserved sense of familiarity in patient Jon (Gardiner et al., 2006), memorable images under conditions of memory decline are associated with diminished autonoetic awareness. However, it may well be possible that preserved components of the hippocampal cortical circuitry may still allow autonoetic awareness to accompany preserved memorability. This alternative is especially plausible given that degeneration seems to affect specific representations more than others. Indeed, high memorability often but not always is related to autonoetic consciousness of the retrieved material (Broers and Busch, 2020). In that sense, we see the experiential nature of memories and the content of memories as two scales (whether orthogonal or closely linked to each other) on which memory function needs to be evaluated.

A thorough understanding of the relationship between memorability and autonoetic consciousness allows us to gain insight into the daily experience with Alzheimer's dementia, as has been done within the area of autobiographical memory. Accessing sensory and perceptual episodic aspects of an event is also related to a sense of self (Conway, 2001; Conway and Pleydell-Pearce, 2000; Piolino et al., 2009; Muireann Irish et al., 2011). In Alzheimer's disease the self becomes more abstract as highly personal semantic information prevails (Addis and Tippett, 2004; Strikwerda-Brown et al., 2019; Caddell and Clare, 2010; Martinelli et al., 2013). The identification of memorable and autonoetic aspects within a memory may serve to boost the subjective sense of self (Prebble et al., 2013).

While our paper is focused on the nature of episodic memories, related questions could also be raised about semantic memories. In fact, as we pointed out above, the interaction between episodic and semantic aspects may contribute to variability in memorability and semantic features remain among those that need to be explored as rendering an image memorable. The research on memorability is still in its infancy, and it will be interesting to address the question of whether memorability applies to semantic memories and their impairment patterns in neurodegenerative conditions as well. While many mechanisms may apply to both episodic and semantic memory and both types of memory closely interact (Renoult et al., 2019; Tulving and Markowitsch, 1998), we still believe that episodic memory may be special in giving rise to autonoetic consciousness (LeDoux and Lau, 2020). Clarifying the relationship between memorability and autonoetic consciousness will thus also contribute to our understanding of the differences between semantic and episodic memory regarding memorability.

Note that many studies already investigate conditions that make memories stick (under healthy and pathological conditions), whether it is the emotional state, a personally meaningful cue or the extent that the episodic elements are unitized (e.g. Bastin et al., 2013; Cooper et al., 2019; Diana et al., 2011; Kwan et al., 2016; Naveh-Benjamin et al., 2002; El Haj et al., 2020, 2012; El Haj et al., 2018; El Haj and Antoine, 2017b; Hayes et al., 2007 and see Yonelinas, 2002 for factors that enhance recognition). These aspects may only partially explain memorability, as memorability is not only context- and task-independent but also experience-independent and similar across people (Bainbridge, 2020). The memorability of a stimulus for healthy individuals can even be predicted by computational algorithms (Needell and Bainbridge, 2021). We earlier described however that certain esthetic aspects may contribute partially as much as the element's composition, presumably affecting possibilities for unitization. Specific memory assessments that account for the representational nature (e.g. drawings as in Bainbridge et al., 2019b; Morgan et al., 2019 or digital memory rebuilds as in Cooper et al., 2019) instead of assessments focused on experimental nature (like the Remember/Know paradigm) may help to investigate how conditions of memory enhancement relate to intrinsic memorability.

We are not the first to call for a content-specific investigation of memory capabilities at a representational level. As indicated already, within autobiographical memory research, it is standard to evaluate the personal past by treating memories as a conglomeration of different types of information that all need to be evaluated separately for a comprehensive memory profile (Levine et al., 2002). Certain aspects of an autobiographical memory or episode can be preserved while other content is impaired and these nuanced memory profiles are consistent within disease groups (Irish et al., 2011a,b). Recent proposals leave categorical approaches to memory content behind and emphasize that a memory is formed and stored in representations of different dimensionalities and levels of abstraction within the functional architecture of memory (Andermane et al., 2021; Brunec et al., 2018; Ekstrom and Yonelinas, 2020; Irish and Vatansever, 2020; Renoult et al., 2019). Different implications of these abstract to specific gradients in memory representations are emphasized. For instance, the amount of semantic versus episodic aspects in a memory is determined by the position of the respective memory representation on a continuum of more or less contextualization (Irish and Vatansever, 2020) and by the need to access specific details versus gist information (Ekstrom and Yonelinas, 2020; Renoult et al., 2019), extending beyond a dichotomy between semantic and episodic memories (Renoult et al., 2019). As the coarseness or precision of representations is rooted in the functional anatomical architecture of memory (Andermane et al., 2021; Brunec et al., 2018; Ekstrom and Yonelinas, 2020; Irish and Vatansever, 2020; Yonelinas, 2013), partial dysfunction of neural substrates does not cause the full memory to fade but rather to become fragmented memories that draw on remaining representations (Ekstrom and Yonelinas, 2020). Many aspects of the above mentioned representational accounts came together in a recent proposal postulating that episodic memories may fade in a fragmented manner that is compatible to our proposal (Andermane et al., 2021). They elaborate on distinct behavioral findings regarding the forgetting of episodic representations. While item representations seem to fade gradually over time, higher-order episodic representations like narratives seem to be forgotten rather holistically. The underlying representational architecture of episodic memories that we outlined above provides a potential explanation. It will be an exciting avenue for future research to investigate memorability in the light of that concept and link the findings to clinical observations.

The unique angle that our current proposal takes is that we refer to the intrinsic memorability of memories. The functional underpinnings of memorability seem to come into play during encoding and during early phases of molecular synaptic consolidation and are not subject to (systems) consolidation mechanisms (Bainbridge and Rissman, 2018; Mohsenzadeh et al., 2019). Notably some items remain memorable or forgettable, even when memories fade over time (Isola et al., 2014) and memorability is highly specific, even varying within stimulus categories (e.g. Bainbridge et al., 2013; Bainbridge, 2017). Thereby our perspective is fundamentally different from the previous accounts where memories are shaped over time and strongly influenced by task demands (Andermane et al., 2021; Ekstrom and Yonelinas, 2020; Levine et al., 2002; Renoult et al., 2019) with effects on general categories of mnemonic content (e.g. Irish and Vatansever, 2020; Levine et al., 2002; Strikwerda-Brown et al., 2019). That said, however, the memorability of items may be influenced by specific retrieval tasks (Bainbridge et al., 2019b; Broers and Busch, 2020) – an observation that needs further investigation. Finally, we emphasize that memorability is related to recent episodic memories. As indicated in the beginning, our opinion paper is a specific call to reconsider the assessment of recent episodic memory and on mnemonic material that may serve to identify Alzheimer's disease in preclinical and prodromal stages. Likewise, there is also increased interest in the assessment of autobiographical memory towards individual daily life memories (Palombo et al., 2018) and it will be important to consider how these two approaches can be linked to each other. Our observations together with particularly the recent perspective on forgetting by Andermane and colleagues (2021) show that it is now timely to investigate the fragmented nature and content-centric aspects of recent episodic memories and their decline.

## 6 Conclusion

Endel Tulving's episodic memory framework inspired decades of research on the experiential nature of memories. The recent findings on episodic memory decline and content-specific processing routes of mnemonic information support an extension of this framework by the concept of memorability. In Alzheimer's disease, memories may not fade unitarily but in a content-specific manner, mirroring affected cortical regions and presumably leading to islands of recollection. Together with the experiential nature of memory, a new focus on the content of fading recent episodic memories may allow us to reveal yet another layer of human memory function and get closer to understanding this miracle of human nature.

## Credit author statement

**Xenia Grande:** Conceptualization, Writing – Original Draft, Writing – Review & Editing; **David Berron:** Writing – Review & Editing; **Wilma A. Bainbridge:** Writing – Review & Editing; **Anne Maass:** Writing – Review & Editing; **Emrah Düzel:** Conceptualization, Writing – Review & Editing, Funding acquisition.

## Declaration of competing interest

David Berron and Emrah Düzel are scientific co-founders of neotiv GmbH (Magdeburg, Germany).
